# Impulsive delayed reward discounting as a genetically-influenced target for drug abuse prevention: a critical evaluation

**DOI:** 10.3389/fpsyg.2015.01104

**Published:** 2015-09-01

**Authors:** Joshua C. Gray, James MacKillop

**Affiliations:** ^1^Department of Psychology, University of Georgia, Athens, GA, USA; ^2^Peter Boris Centre for Addictions Research, McMaster University/St. Joseph’s Healthcare Hamilton, Hamilton, ON, Canada

**Keywords:** substance use disorders, drug abuse, addiction, behavior economics, delayed reward discounting, behavioral economics, intertemporal choice

## Abstract

This review evaluates the viability of delayed reward discounting (DRD), an index of how much an individual devalues a future reward based on its delay in time, for genetically-informed drug abuse prevention. A review of the literature suggests that impulsive DRD is robustly associated with drug addiction and meets most of the criteria for being an endophenotype, albeit with mixed findings for specific molecular genetic influences. Several modes of experimental manipulation have been demonstrated to reduce DRD acutely. These include behavioral strategies, such as mindfulness, reward bundling, and episodic future thinking; pharmacological interventions, including noradrenergic agonists, adrenergic agonists, and multiple monoamine agonists; and neuromodulatory interventions, such as transcranial magnetic stimulation and transcranial direct current stimulation. However, the generalization of these interventions to positive clinical outcomes remains unclear and no studies to date have examined interventions on DRD in the context of prevention. Collectively, these findings suggest it would be premature to target DRD for genetically-informed prevention. Indeed, given the evidence of environmental contributions to impulsive DRD, whether genetically-informed secondary prevention would ever be warranted is debatable. Progress in identifying polymorphisms associated with DRD profiles could further clarify the underlying biological systems for pharmacological and neuromodulatory interventions, and, as a qualitatively different risk factor from existing prevention programs, impulsive DRD is worthy of investigation at a more general level as a novel and promising drug abuse prevention target.

## Introduction

Excessive use of addictive drugs is both widespread and onerous, contributing to to approximately 22% of deaths and costing more than $500 billion annually in the United States (Mokdad et al., 2004; Uhl and Grow, 2004). A high priority for reducing the burden of addictive disorders is to translate knowledge of the underlying risk factors for addiction into prevention and early intervention approaches. Numerous factors influence the probability of initiation and progression of drug use, but one well established domain is genetic variation, which is estimated to contribute approximately half of the liability for developing drug addiction (Goldman et al., 2005; Agrawal and Lynskey, 2008). From a theoretical perspective, aligning prevention efforts to address genetic risks has very high potential, as it would focus on important etiological variables that are not currently considered from a prevention perspective and would seek to assist individuals who are constitutionally at elevated risk. It would be a form of personalized medicine, but at the level of prevention. Ideally, genetically-informed prevention programming would go one step further and would target the specific ways that genetic variation give rise to drug abuse risk, the biopsychosocial mechanisms of risk. By permitting very early risk identification and the delivery of maximally relevant prevention programming, prevention strategies that are specifically tailored to genetically-influenced risk mechanisms would have the potential to have a major impact.

At a behavioral level, an increasingly well-established risk factor for drug abuse is impulsive decision making, specifically, the propensity to select an immediate reward at the expense of greater future rewards. This form of impulsivity is typically referred to as delayed reward discounting (DRD) or capacity to delay gratification, and has also been increasingly linked to genetic influences. The link to genetics in turn suggests that impulsive DRD may be a viable candidate for genetically-informed prevention. In practice, what this means is that individuals with genetic profiles associated with more impulsive DRD would pre-emptively receive programming to reduce preferences for immediate gratification and, ultimately, to reduce the probability of subsequent drug abuse. This would be a radically different strategy from current prevention efforts and could be very powerful, both for preventing drug abuse and a number of adverse health outcomes. However, it is also lofty prospect that is highly contingent on a number of relationships being empirically robust.

The goal of the current review is to concretely evaluate the existing literature on the prospects of DRD as a genetically-informed prevention target. The review has four goals: (1) to introduce DRD as a behavioral characteristic and review its association with drug abuse; (2) to review the evidence suggesting DRD is an endophenotype (i.e., a genetically-influenced mechanism of risk for addictive disorders); (3) to review candidate intervention approaches for reducing impulsive DRD; (4) to critique the extent to which the preceding sections “connect the dots” to make an compelling argument for such an approach.

## Methodology

To conduct our review, we examined the published literature using the Public Library of Medicine (PubMed) and PsychINFO databases. Specifically, we examined individual empirical articles and reviews that addressed DRD in the context of drug abuse, behavioral genetics, and manipulations or interventions that reduce impulsive DRD. The review included studies on DRD in both humans and non-human animals, but, given that DRD is generally independent of other measures of impulsivity (e.g., MacKillop et al., 2014), we did not include studies of other domains. Articles in the two major domains of the study (genetic influences on DRD and strategies for reducing DRD) were critiqued in detail and the accompanying cited works were used to identify other potentially relevant studies. However, a specific search protocol using a discrete set of search terms was not implemented, meaning that this article is more appropriately considered a critical review of the literature, but not a formal systematic review (e.g., Khan et al., 2003).

## Delayed Reward Discounting and Drug Abuse

**FIGURE 1:**
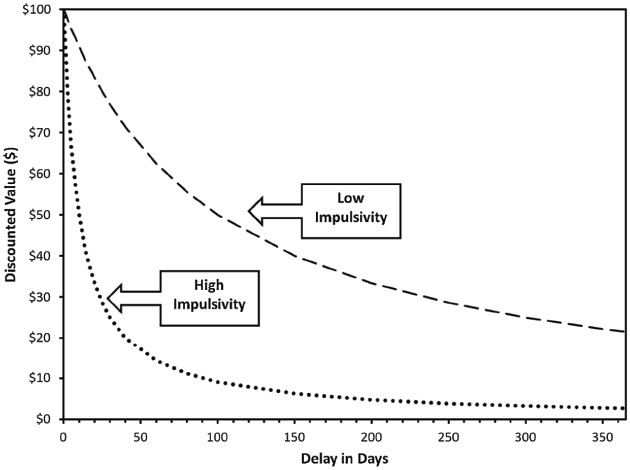
**Prototypic hyperbolic delayed reward discounting curves reflecting the discounted subjective value of $100 delayed from 1 day to 1 year The curves reflect the points at which the smaller immediate reward is equal in value to the $100 delayed reward.** For example, at a delay of 100 days, $100 has lost ∼50% of its nominal value for the low impulsivity profile and ∼90% of its nominal value for the high impulsivity profile. Figure from MacKillop (2013).

Delayed reward discounting is typically assessed using decision-making tasks posing choices between a smaller monetary reward that is immediately available and a larger delayed reward after an intervening delay. By varying the reward amounts and the delay length, an overall characterization of temporal discounting rates can be generated. Figure 1 provides illustrative discounting temporal discounting curves for $100 available in the future versus smaller amounts available in the present. Quantitative indices of discounting are typically generated either using non-linear regression to derive an individual’s temporal discounting function (i.e., *k*) or generating the area under the curve (AUC; Myerson et al., 2001). The *k* index reflects the slope of the hyperbolic discounting function and AUC reflects the overall volume of the discounting curve. The two are strongly inversely correlated; steeper discounting curves have high *k* values and low AUCs. Both indices have their own advantages and disadvantages, with the primary difference being that *k* makes implicit assumptions about the hyperbolic form of the discounting function, whereas AUC is theory-free and does not make assumptions about the specific form of the curve (Myerson et al., 2001). Although often administered for hypothetical outcomes, a number of studies suggest that individuals respond similarly on versions of the task in which they are provided with an actual monetary reward based on their responses (Johnson and Bickel, 2002; Madden et al., 2003; Bickel et al., 2009; Lawyer et al., 2011). Furthermore, studies have demonstrated robust test-retest reliability, with comparable stability to personality traits (Beck and Triplett, 2009; Kirby, 2009; Odum, 2011). Money is the most commonly used commodity and has a number of advantages (e.g., generality of relevance, meaningfulness of discrete units), but other commodities, including addictive drugs, can be assessed using DRD paradigms. Indeed, some of the earliest work in this area used the now famous “marshmallow test” in which children choose between one marshmallow immediately available after the experimenter leaves the room or two marshmallows if they wait for the experimenter to return (Metcalfe and Mischel, 1999).

Individuals who strongly prefer immediate over delayed rewards of larger value are said to exhibit impulsive discounting of delayed rewards. Impulsive DRD is associated with earlier age of addiction onset (Dom et al., 2006), dependence on multiple classes of drugs (e.g., tobacco, alcohol, cocaine; MacKillop et al., 2011), and treatment response (MacKillop and Kahler, 2009; Sheffer et al., 2014). Furthermore, a meta-analysis synthesized previous literature on DRD in relation to addictive behavior by comparing levels of DRD between criterion (addicted) groups and control groups (MacKillop et al., 2011), finding consistent evidence of significantly more impulsive DRD in criterion groups, with a medium effect size across studies (*d* = 0.58).

Although debate has arisen regarding the extent to which DRD is a cause or consequence of addiction (or whether they are both influenced by a third variable), there is increasing evidence that DRD preferences at least partially predates the development of addiction. Two retrospective studies have identified that more impulsive DRD predicts earlier onset of alcohol use (Kollins, 2003) and alcohol use disorder symptoms (Dom et al., 2006). Several subsequent studies have found a link between more impulsive DRD in adolescents and increased substance use and/or misuse over time (Audrain-McGovern et al., 2009; Fernie et al., 2013; Khurana et al., 2013; Kim-Spoon et al., 2014). However, one recent study did not find a consistent connection between DRD and subsequent substance use (Isen et al., 2014). Notably, the null results in this study may be partially attributable to their utilization of an externalizing latent factor that included a broader spectrum of externalizing behaviors than simply drug use and misuse (e.g., disinhibited, delinquent, and aggressive behavior). In sum, DRD is a well-validated behavioral measure of impulsivity, is consistently associated with addictive behavior, and is an etiological risk factor, predating alcohol and tobacco use and misuse.

## Delayed Reward Discounting as a Drug Abuse Endophenotype

Although approximately ∼50% of all variance in addictive disorders is genetic risk (Goldman et al., 2005; Agrawal and Lynskey, 2008), little variance has been consistently accounted for by molecular genetic studies. In fact, candidate gene studies (assessing associations with a small number of variants in a limited number of genes) and genome-wide association studies (assessing associations with hundreds of thousands of variants across the genome) have both identified variants which are inconsistently replicated and exhibit small effect sizes (Goldman et al., 2005; Treutlein and Rietschel, 2011). This gap between high levels of heritability and specific variants of inconsistent and small effects is referred to as the “missing heritability problem” (Turkheimer, 2011). Several potential factors contribute to this issue, but perhaps two are most notable: (1) addictive disorders are highly polythetic (i.e., hundreds of combinations of symptoms can produce the same diagnosis); and (2) addictive disorders are “too far” from the genes, meaning that the proximal consequences of genetic variation may be only distantly related to the proximal risk factors for drug abuse. As a result of these obstacles, an endophenotype approach has been proposed, shifting the focus to narrower phenotypes that are putatively determined by a more limited number of genes and are more specifically associated with the disorder of focus. Endophenotypes are also intended to be mechanistically informative about the nature of genetic influences. Given both links to genetics and mechanisms of risk, endophenotypes are the natural intervention targets in the context of genetically-informed prevention.

Importantly, a number of criteria have been increasingly accepted as defining an endophenotype. These comprise evidence of the following: (1) association with the illness, meaning a link with the condition of interest; (2) heritability, meaning evidence that the characteristic is influenced by genetics; (3) state independence, meaning the characteristic is present when the disease is not (and is not simply a symptom of the condition); (4) present in non-affected family members at higher rates than the general population, further indicating its genetic basis; and (5) co-segregation with the psychiatric illness in families, further indicating association (Gottesman and Gould, 2003).

For DRD, the first of these criteria was addressed above, in the links between the behavioral characteristic of DRD and drug abuse. Shifting to the heritability of DRD, there is robust evidence from animal and human studies. Animal studies are particularly useful for assessing heritability of traits because they allow researchers to control all aspects of the environment. The reduction in environmental variability enables isolation of the effects of genetic variability. In animal studies, researchers typically compare behaviors across inbred strains that are isogenic (i.e., entirely or nearly genetically identical; Falconer et al., 1996). In the first rodent study of DRD heritability, approximately 16% of variability in DRD rates was attributable to between-strain differences in mice (Isles et al., 2004). Studies of Lewis and Fischer rodents reared in identical environments also identified systematic differences in discounting across strains that are attributable to genetic differences (Anderson and Woolverton, 2005; Madden et al., 2008; Stein et al., 2012). Finally, in a recent study, the estimated heritability across eight strains was between 43 and 66% (Richards et al., 2013). Overall, these studies largely found robust differences in DRD across rodent strain, suggesting substantial heritability of DRD.

To date, four human studies have assessed the heritability of delay discounting and all four identified evidence of heritability. Early adolescent twins were found to have genetic influences on DRD at ages 12 (30%) and 14 (51%, Anokhin et al., 2011). Additionally, in a sample of 17-year-old twins, strong evidence of heritability was found in two different DRD phenotypes (47–51%, Isen et al., 2014; Sparks et al., 2014). Most recently, Anokhin et al. (2015) assessed DRD in a sample of twins and found significant heritability of both DRD indices (AUC: 46 and 62%; *k*: 35 and 55% at age 16 and 18 respectively). The trend of increasing genetic influence in later adolescence is likely attributable to ongoing adolescent brain maturation of prefrontal regions implicated in intertemporal choice (Carter et al., 2010; Steinberg, 2010; Peters and Büchel, 2011; Luo et al., 2012). Taken together, both animal and human studies suggest that DRD is heritable and possesses similar rates of heritability as addiction phenotypes (i.e., ∼50%).

In the domain of family history, rodent studies support the presence of elevated levels of DRD in non-affected family members (as compared to the general population). Specifically, three studies to date of alcohol-naïve rodents selectively bred for high- or low-alcohol preference, found that high-alcohol preferring subjects exhibited an increased rate of DRD of sucrose rewards (Wilhelm and Mitchell, 2008; Oberlin and Grahame, 2009; Perkel et al., 2015). Notably, one study did not find a difference in DRD of sucrose rewards between high- and low-alcohol preferring rodents (Wilhelm et al., 2007). Nonetheless, the majority of evidence suggests that heritability for alcohol abuse susceptibility overlaps with heritability for DRD preference, and that in subjects susceptible to alcohol abuse, impulsive DRD is present prior to alcohol exposure.

While human research has been mixed regarding the presence of DRD at elevated rates in non-affected family members, earlier studies suffered from significant methodological issues (most notably, small sample size; e.g., Crean et al., 2002; Petry et al., 2002; Herting et al., 2010). A more recent highly-powered study found that in 298 healthy young adults (age *M* = 23), those with a family history positive for alcohol or other drug use disorders had higher rates of DRD (Acheson et al., 2011). Furthermore, the study found that impulsive DRD was significantly associated with having more parents and grandparents with alcohol and drug use disorders. Similarly, Dougherty et al. (2014) found that in 386 non-affected youth (ages 10–12), those with family histories of alcohol or other drug use disorders had higher rates of DRD. These findings suggest that in studies with adequate power and a thorough assessment of family history of substance use disorders, there is evidence that non-affected family members of individuals with substance use disorders possess higher rates of DRD than the general population. Similarly, this body of research suggests that given the overlap in heritability of drug abuse and impulsive DRD, there is likely an overlap of specific genetic loci conferring risk for drug abuse and for DRD.

Relatively recent efforts have been made to determine the molecular genetic basis of DRD, primarily within dopaminergic genes. Currently, findings primarily suggest the involvement of the single nucleotide polymorphisms (SNPs) from *COMT* (rs4680) and *ANKK1* (rs1800497), and the exon 3 variable number of tandem repeats (VNTR) polymorphism in *DRD4*, genes which are all implicated in dopamine neurotransmission (Boettiger et al., 2007; Eisenberg et al., 2007; Paloyelis et al., 2010; Gianotti et al., 2012; Smith and Boettiger, 2012; Gray and MacKillop, 2014). Regarding rs4680, four studies found an association between possession of the G allele and impulsive DRD in adults (Boettiger et al., 2007; Gianotti et al., 2012; Smith and Boettiger, 2012; MacKillop et al., in press), one found an association of A/A with impulsive DRD in young adults (Paloyelis et al., 2010), and another found no association (Gray and MacKillop, 2014). The A/A genotype of rs4680 is associated with a reduction in levels of catechol-*O*-methyl transferase enzymatic activity (an enzyme implicated in dopamine catabolism), which leads to higher levels of dopamine primarily in the prefrontal cortex (Weinshilboum et al., 1999; Chen et al., 2004; Tunbridge et al., 2004). Gianotti et al. (2012) found that reduced activity in the left dorsal prefrontal cortex (dPFC) during a resting state paradigm mediates the effect of the G allele on impulsive DRD (also see Boettiger et al., 2007). This suggests that the G allele of rs4680 reduces baseline dPFC engagement via reduced dopamine availability, leading to more impulsive decision making. The dPFC does indeed appear to be strongly implicated in impulsive decision making as it is known to impact self-control processes (Gianotti et al., 2009; Knoch et al., 2010) and the dorsolateral prefrontal cortex (dlPFC) has been shown to affect DRD rates when stimulated transcranially (discussed below). Future studies with large healthy populations are required to verify which genotype is of greatest risk and examine moderators (e.g., age effects), as one recent study’s findings suggest a U-shape curve between dopamine levels and DRD performance (i.e., too much or too little dopamine yields impulsive DRD; Smith and Boettiger, 2012). Nonetheless, current research supports a relationship between *COMT* (rs4680) and DRD rates via dPFC dopamine levels.

The T allele of rs1800497 has been associated with DRD in two studies (Eisenberg et al., 2007; MacKillop et al., in press), and not associated in two others (Kawamura et al., 2013; Gray and MacKillop, 2014). However, considerable heterogeneity in sample demographics (e.g., healthy college students, weekly gamblers, healthy adults) and sample sizes (between 91 and 195 participants) may explain the mixed findings. The role of the rs1800497 SNP is less well understood because it is technically in the *ANKK1* gene, near the *DRD2* gene. However, rs1800497 is in high linkage disequilibrium with SNPs from multiple genes in this region (*NCAM1-TTC12-ANKK1-DRD2*, Mota et al., 2012) and is associated with dopamine D_2_ receptor density (Pohjalainen et al., 1998; Jönsson et al., 1999; Savitz et al., 2013). Regardless of the specific mechanism of influence of rs1800497, its association with dopamine availability and with multiple addictive genotype influences (for a review see Ma et al., 2014) suggests it should be investigated further in relation to DRD rates.

*DRD4* VNTR influences intracellular levels of cyclic adenosine monophosphate to primarily impact dopamine response in the prefrontal cortex, however, the specific downstream biochemical impact of different variants of *DRD4* VNTR remains relatively unclear (Oak et al., 2000) and recent studies have examined the role of rare variants rather than length of repeats (e.g., Tovo-Rodrigues et al., 2012; Michealraj et al., 2014). *DRD4* VNTR and DRD has been explored in several studies, with mixed findings, and appears to have a more context dependent relationship with DRD rates. For example, one study found the combination of the long form of *DRD4* VNTR and the T allele of rs1800497 to be associated with significantly higher DRD rates (Eisenberg et al., 2007), and a second study found increased DRD rates in low socioeconomic status (SES) long form carriers versus decreased DRD rates in mid-to-high SES long form carriers (Sweitzer et al., 2013). In addition, studies have reported a direct negative relationship between the long from and decreased DRD rates (Gray and MacKillop, 2014) and no direct association (Eisenberg et al., 2007; Garcia et al., 2010; Paloyelis et al., 2010; Sweitzer et al., 2013). However, the existing studies have varied widely in sample composition (e.g., healthy college students, adolescents with attention deficit hyperactivity disorder [ADHD]) and size (ranging from 68 to 546). It will be important for future studies to continue to explore the potential of *DRD4* VNTR as a differential susceptibility gene (see Bakermans-Kranenburg and van Ijzendoorn, 2011) in order to determine whether the relationship between DRD and polymorphisms of varying length or rarity is contingent upon other genes or environmental stressors.

Despite some promising findings regarding the role of *COMT*, *DRD2*, and *DRD4*, the associations require consistent replication and the effect sizes have been relatively small. Nonetheless, current empirical findings and theory suggest a central involvement of dopamine functioning as well as possible interactions among serotonin and dopamine systems on DRD performance (Winstanley et al., 2005; Simon et al., 2013). Greater exploration of other systems related to reward processing as well as genome-wide association studies are a priority for future research. Identification of robust genetic correlates of DRD would provide insights into the neurobiological causes of variation, identifying targets for possible pharmacological and neuromodulatory interventions.

Taken together, DRD is relatively well supported as an endophenotype for addictive disorders, although the identification of specific polymorphisms responsible for variation is nascent. The initial molecular genetic studies suggest that dopamine transmission plays an important role in DRD, yet in almost all cases, the candidate loci were the ‘usual suspects’ (i.e., loci tested most frequently for associations with addictive behavior and other externalizing psychopathology). Future work that establishes the robustness of these findings and expands the genomic perspective will be essential.

## Interventions Targeting Delayed Reward Discounting

Several experimental manipulations have been examined for reducing high rates of DRD, and can be broadly classified into three domains: behavioral interventions, pharmacological interventions, and neuromodulatory manipulations using transcranial stimulation of specific brain regions.

### Behavioral Interventions

The earliest research exploring the link between distraction and DRD was conducted on preschool age children (3–5 years old) who underwent the aforementioned marshmallow test. In this early work, when encouraged to think of other things or play with toys, the children more frequently waited longer for the delayed reward (Mischel and Ebbesen, 1970; Mischel et al., 1972). Similar findings have been identified in animal studies (Grosch and Neuringer, 1981; Evans and Beran, 2007). This is thought to operate similarly to distraction manipulations that lead to more effective resistance to food or drug cravings in susceptible individuals (e.g., Versland and Rosenberg, 2007; Van Dillen et al., 2013; Murphy and MacKillop, 2014). However, it remains unclear whether distraction techniques can offer long-term (rather than merely temporary) disruption of immediate reward pursuit (see Ashe et al., 2015).

The converse of distraction-based techniques is a mindfulness approach which seeks to encourage non-judgmental and objective monitoring of one’s own thoughts and behaviors in an effort make well considered, unimpulsive decisions (Marlatt, 2002). For example, one study employed a brief 60–90 min training based on Acceptance and Commitment Therapy (Hayes et al., 2011; Morrison et al., 2014). In this training session, subjects discussed internal barriers to healthy decision making with a therapist and engaged in several exercises designed to aid the participant in observing their emotions and learning to act on values rather than feelings. Participants who were engaged in this training procedure exhibited decreases in DRD, whereas waitlist controls did not. Brief mindfulness-based interventions have been adapted for obese individuals and have shown efficacy for reducing DRD of food items (Hendrickson and Rasmussen, 2013). In a similar spirit of priming mindfulness, Berry et al. (2014) found that preemptive and concurrent visual exposure to natural environments (e.g., mountains), led to approximately a 50% reduction in DRD compared to decisions made during exposure to built environments (e.g., buildings) and control environments (e.g., triangles). All of the work in this area has focused on acute outcomes and it will be important for future studies to explore longitudinally how mindfulness training may influence long-term decision making patterns.

Beyond distraction and mindfulness, a wide variety of other behavioral techniques have been applied to reduce impulsive discounting. For example, a small number of early studies employed a fading procedure with pigeons that gradually increased the delay between the small reinforcer and the larger delayed reinforcer, which yielded an increased selection of the delayed reward (Mazur and Logue, 1978; Logue et al., 1984). Similar studies have been conducted primarily in children with conditions associated with impulsivity (e.g., mental retardation, autism, ADHD) and have found reductions in DRD (Schweitzer and Sulzer-Azaroff, 1988; Dixon et al., 1998; Fisher et al., 2000). In the context of these experiments, the participants were offered a small immediate reinforcer or a large reinforcer that was contingent on engagement in a target behavior (e.g., staying seated) for a required duration. The duration for performing the target behavior was gradually increased overtime and the children typically showed increasing ability to maintain this behavior for extended periods of time in order to obtain the larger reinforcer. However, sample sizes ranged from 3 to 6 participants and the applicability of this technique to healthy adolescents (a typical target sample for drug abuse prevention) or substance using adults is relatively low.

Another method of reorienting individuals toward larger delayed rewards is “reward bundling,” or grouping a series of DRD choices into a single decision. For example, for the “reward bundling” condition, one recent study informed participants that if they choose a smaller sooner reward then they will receive that reward every 2 weeks after that for 6–10 weeks, and if they choose a larger delayed reward in 10 days then they will receive that reward every 2 weeks after that for 6–10 weeks (Hofmeyr et al., 2011). This makes theoretical sense, as orienting individuals to considering a series of consequences of a pattern of decision making (as opposed to a consequence derived from a single choice) may increase their consideration of avoiding a sum of reduced rewards by choosing to favor larger greater rewards. For example, if the choice to get intoxicated now at the cost of feeling good tomorrow were to entail commitment to this same choice every day for the next week, the value of the larger delayed reward relative to the smaller sooner reward would presumably increase (Monterosso and Ainslie, 2007). Bundling has been supported empirically by several laboratory studies involving animals and humans (Kirby and Guastello, 2001; Ainslie and Monterosso, 2003; Hofmeyr et al., 2011; Stein et al., 2013). The most recent human study, conducted by Hofmeyr et al. (2011), found that smokers, but not non-smokers, were particularly susceptible to the reward bundling manipulation. This suggests that those who are more susceptible to addictive behavior may be in greater need of and more responsive to interventions that challenge them to consider the long-term aggregation of rewards. Relatedly, a study found that in cocaine and/or alcohol outpatient substance users, an intervention comprised of individual counselor-facilitated training in monthly budgeting, which focused on long term goals and limited short-term spending, led to a decrease in both DRD and cocaine use (Black and Rosen, 2010).

Another strategy for modifying discounting is episodic future thinking, which is a method of increasing future orientation by prompting individuals with autobiographical, emotional, and circumstantial details that are expected to occur at specified delays in the future (Atance and O’Neill, 2001). For example in two fMRI experiments, Peters and Büchel (2010) found that when delays were paired with events the subjects were likely to engage in during that time (e.g., “20€ now or 35€ in 45 days (vacation Paris)”), subjects were more likely to choose the delayed rewards than when rewards were not presented with these tags. This finding has been replicated in three additional studies (Benoit et al., 2011; Daniel et al., 2013a,b). Most recently, a study found that episodic future thinking is not dependent on positive affect induction for its effects rather, even neutral-valenced events shift time perspective to reduce DRD (Lin and Epstein, 2014). Using a conceptually similar strategy, one investigation conducted four studies utilizing virtual reality to display computerized renderings of participants’ future selves, and in all cases they found that those who interacted with their virtual future selves had reduced DRD (Hershfield et al., 2011). This represents a promising method for engaging individuals in greater imagination of their future in order to reduce DRD.

From a more purely cognitive standpoint, a phenomenon that has been demonstrated in several studies is framing effects, or the tendency of DRD to fluctuate in relation to the specific wording of the delay. Read et al. (2005) first demonstrated that when delays are framed as calendar dates (e.g., on December 5), discount rates tend to decrease and the shape of the discount function becomes more linear (less hyperbolic). Other studies have had similar findings (LeBoeuf, 2006; Klapproth, 2012; DeHart and Odum, 2015). Notably, other variables involved in question framing have been shown to either decrease DRD, such as presenting participants with an explicit zero paired with the options (e.g., “[A] $5.00 today and $0 in 26 days OR [B] $0 today and $6.20 in 26 days”; Magen et al., 2008). A common element across all of these formats is that they seem to increase the salience of the delay by framing the specific date (possibly increasing the perceived likelihood of actually receiving the reward; see Patak and Reynolds, 2007), increasing attention to the notion that they will receive “$0” at the delay if they select the immediate reward.

Finally, strengthening the elementary cognitive processes that subserve DRD decision making represents a further strategy for reducing this form of impulsivity. Two studies have been conducted to explore the extent to which working memory training can improve overall executive functioning capabilities as a way to decrease DRD and improve overall decision making capabilities. The first study randomized a small number of individuals (*N* = 27) into a training condition and a matched control condition (Bickel et al., 2011). The working memory training used a computer program consisting of several challenges (e.g., recalling a sequence of digits forward or backward) administered 4–15 times over the course of approximately 25 days. In the control condition, participants were exposed to the same set of stimuli, but were provided with the answers so that they did not need to engage their working memory. The study found that the working memory training group significantly decreased discounting rates by approximately 50%, whereas the control group exhibited no significant reductions in DRD. A recent study did not replicate the connection between working memory training and reduced DRD in a rodent model (Renda et al., 2015), although major methodological differences were present (e.g., species, type of task, prior substance use). Ameliorating delay discounting via working memory is at an early stage but has considerable promise.

### Pharmacological Interventions

Several studies have tested the efficacy of dopamine (DA) agonists (e.g., amphetamine) and DA-norepinephrine (NE) agonists (e.g., methylphenidate) for reducing DRD. Frequently prescribed to individuals with ADHD, amphetamine and methylphenidate are thought to increase executive functioning capacity by facilitating transmission of catecholamines in critical regions (Bidwell et al., 2011). Studies have found that both amphetamine and methylphenidate typically reduce DRD in rat models (Cardinal et al., 2000; Adriani et al., 2004; van Gaalen et al., 2006; Bizot et al., 2011), however, null or even opposite effects have occasionally been detected when varying methodology (e.g., rearing environments, signaled or unsignaled rewards; Cardinal et al., 2000; Perry et al., 2008). Of the human studies that have been conducted, one found that amphetamine decreased DRD in healthy adults (de Wit et al., 2002), and the others found that methylphenidate decreased DRD in a sample of adults with a criminal background (Pietras et al., 2003) and in a sample of children with ADHD (Shiels et al., 2009). Despite the promise of these human studies, the therapeutic use of these substances in reducing DRD must be balanced with their high abuse potential in individuals without ADHD (Kollins, 2007).

Additional compounds have been examined for efficacy in reducing DRD, including compounds with less direct and concentrated effects on DA availability, such as NE agonists (e.g., atomoxetine), adrenergic agonists (e.g., guanfacine), and multiple monoamine agonists (e.g., modafinil; Wilens, 2006). These substances are of particular interest because they uniformly exhibit minimal abuse potential (Malcolm et al., 2002; Muir and Perry, 2010; Upadhyaya et al., 2013). Among these three compounds, atomoxetine has been studied most extensively, but only in rodent models to date. Early research by Robinson et al. (2008) found that atomoxetine significantly decreased several forms of impulsivity, including DRD. Similarly, Bizot et al. (2011) found that subjects given atomoxetine were more likely to select the large but delayed reward. However, other studies have found atomoxetine increased DRD in healthy rodents (Broos et al., 2012), or had no effect on DRD in healthy (Baarendse and Vanderschuren, 2012), spontaneously hypertensive (an animal model for ADHD; Turner et al., 2013), and cocaine-withdrawing rodents (Broos et al., 2014). In the latter study, despite no changes in DRD, the rodents were less likely to readminister cocaine at 1 and 10 days (Broos et al., 2014). Finally, one study found that chronic atomoxetine treatment during adolescence (but not acute atomoxetine in adulthood) led to a stable decrease in DRD when tested in adulthood, suggesting lasting effects of the atomoxetine in the orbitofrontal cortex (Sun et al., 2012).

Early studies testing effects of guanfacine and modafinil on DRD are promising. One study found that intramuscular guanfacine reduced DRD in rhesus monkeys (Kim et al., 2012) and a second found dose-dependent reduction in DRD in rats when guanfacine was administered locally in the ventral hippocampus (Abela and Chudasama, 2014). In a fMRI study on humans, modafinil was found to decrease DRD in alcohol dependent participants, but yielded no change in healthy control subjects, suggesting that modafinil normalizes DRD decision making in alcohol dependent patients (Schmaal et al., 2014). Moreover, reductions in DRD were accompanied by an enhanced functional connectivity between the superior frontal gyrus and ventral striatum, suggesting more prefrontal control over these choices. It will be important for future studies to continue to examine the effects of these medications on DRD, particularly in humans with and without high levels of DRD, to establish the consistent and stable effects.

### Neuromodulatory Manipulations

Recent efforts have been made to explore the impact of human non-invasive transcranial brain stimulation on DRD, both through magnetic and direct electrical current stimulation. Transcranial magnetic stimulation (TMS) operates by passing electricity through a coil placed near the region of focus. The resulting magnetic field can be used to temporarily modulate brain activity in nearby regions. One group assessed the effect of dlPFC interruption during a DRD task as well as a single item valuation task (i.e., participants rated the attractiveness of 12 single-options taken from the DRD choice set; Figner et al., 2010). They found that left (but not right) dlPFC inhibition increased impulsive responding on the DRD task, but neither region impacted item valuation on the single item valuation task. This suggests that the left dlPFC is critical to self-control (inhibiting responses for salient immediate rewards) rather than in stimulus value appraisal. However, inhibition of the right dlPFC was observed to reduce impulsive DRD in another study (Cho et al., 2010), but only when participants were exposed to a significantly higher frequency and shorter duration of magnetic stimulation than in the study by Figner et al. (2010). Additionally, a subsequent study using positron emission tomography (PET) found that inhibition of the right dlPFC reduced impulsive DRD rates (reducing impulsivity) and disrupted regional cerebral blood flow (rCBF) in the right dlPFC and right rostral PFC leading to diminished correlations between DRD rates and rCBF of these (and other) prefrontal regions (Cho et al., 2012). This suggests that the neural network underlying impulsive decision making is disrupted by right dlPFC inhibition. Finally, also using PET imaging, stimulation of the medial prefrontal cortex has been found to both reduce DRD rates and reduce the level of synaptic dopamine in the striatum (Cho et al., 2015). This is clearly a mixed literature and discrepancies among these findings will need to be reconciled in future studies.

In contrast to TMS, transcranial direct current stimulation (tDCS) operates by directly passing electrical currents to surface electrodes placed on the scalp proximal to the region(s) of interest. One study found no effect of inhibition of left dlPFC and stimulation of right dlPFC on DRD, but did find that when the right dlPFC is inhibited and the left dlPFC is stimulated, impulsive DRD rates increase (Hecht et al., 2013). A second study also found no effects of left dlPFC inhibition and right dlPFC stimulation (Kekic et al., 2014). Clearly, this work is at an early stage and future research should seek to replicate and clarify these findings. Additional priorities include further exploration of the role of anode/cathode placement, electrode size, and current intensity on DRD rates.

Despite early studies showing promise of non-invasive brain stimulation of the prefrontal cortex in improving impulsive decision making, it will be important for future studies to clarify the ideal tool for stimulation (i.e., TMS or tDCS), frequency and intensity of stimulation, and manipulation of left and right prefrontal areas. Although research is nascent, TMS appears to have support both from aforementioned studies and from interventions for related cognitive functions. For example, a recent meta-analysis assessed the efficacy of TMS and tDCS of the dLPFC to improve working memory performance (Brunoni and Vanderhasselt, 2014), a cognitive function that has been linked to DRD performance (Bickel et al., 2011; Wesley and Bickel, 2014). They found that TMS improved response time and accuracy whereas tDCS improved only response times. Several possible mechanisms may explain differences in effects found such as difference in study design, equivalency of “doses,” and better spatial precision of TMS. In addition to identifying optimal stimulation methodology, it will be important for future studies to explore the potential long term effects of transcranial stimulation treatment for DRD.

## Critique of Delayed Reward Discounting as a Genetically-Informed Drug Abuse Prevention Target

**FIGURE 2:**
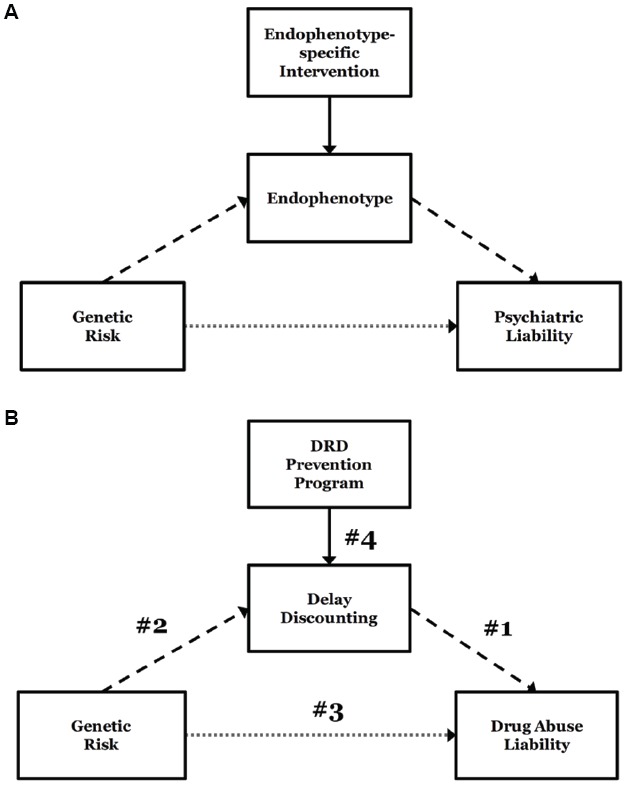
**A framework for considering developing genetically-informed prevention and treatment.** General theoretical framework for an endophenotype approach to addiction genetics, integrating endophenotype-specific treatment approaches (Ray et al., 2010) **(A)**. Specific model of delayed reward discounting (DRD) as an endophenotype of genetic risk for addiction liability **(B)**. Numbers 1–4 reflect the necessary components to establish DRD as a viable genetically-influenced prevention target for drug abuse: (1) a robust relationship between DRD and drug abuse; (2) genetic loci that are reliably associated with delay discounting; (3) possession of risk alleles for DRD risk that are responsible for drug abuse risk; (4) efficacious strategies for reducing impulsive DRD.

In the preceding sections, we reviewed impulsive DRD as a behavioral characteristic, evidence linking it to genetic influences, and evidence that it can be significantly ameliorated using a number of strategies. Here, we consider the assembled findings and the specific question of whether it has promise for genetically-informed drug abuse prevention. To do so, we provide a framework for considering the links that are necessary for coming to that conclusion (Figure 2). The framework is an extension of a previous model for integrating alcohol endophenotypes into treatment development and prospective pharmacotherapies (Ray et al., 2010). As depicted in Figure 2A, we propose that endophenotypes can enhance the prospect of genetically-informed intervention of any kind (prevention or treatment) by identifying genetically-mediated risk mechanisms that both enhance the resolution of risk status and serve as intervention targets. In the first case, endophenotypes (e.g., impulsive DRD) are anticipated to ultimately lead to more reliable identification of risk alleles via more robust relationships with individual genotypes. In the second case, the identification of risk status in the context of a specific mechanism implicitly reveals a candidate intervention target. In other words, endophenotypes have the potential to elucidate both the biological causes of the disorder and provide personalized intervention targets.

Figure 2B lays out the case for DRD, identifying the four links that necessarily comprise an argument for genetically-influenced prevention programming. These can be summarized by the following questions:

(1) Is DRD reliably associated with drug abuse?
(2) Are specific genetic loci reliably associated with DRD?
(3) Are DRD risk alleles also responsible for drug abuse risk?
(4) Are there established strategies for reducing impulsive DRD?

If the empirical support for the links in Figure 2B is reliably present, the case for a genetically-informed DRD prevention strategy would be entirely sound. Where those links are less than robust, however, there remain ambiguities and open questions, and the rationale becomes more debatable.

In light of the preceding sections, it is clear the latter is the case for DRD. In the discounting framework, the strongest link is the first, the association between impulsive DRD and drug abuse. As discussed, this relationship has been observed in an array of different samples with an array of different methods, cross-sectionally and longitudinally. With regard to the genetic linkages (2 and 3), the literature remains at an early stage. As noted earlier, although there is relatively strong evidence that DRD is heritable, there is not a sufficiently strong basis for defining individuals at higher or lower genetic risk based on individual genotypes or multi-locus risk scores at this time. Furthermore, there is very limited evidence that discounting risk alleles indirectly impact addictive behavior; this has only been directly demonstrated in one study (Gray and MacKillop, 2014). Similarly, at the level of link 4, intervention research on DRD remains incipient. Although several methods have been applied to reducing DRD rates, there is limited consensus on the ideal approach or combinations of approaches. Furthermore, many of the methods have not been replicated, examined for prolonged reductions, or tested in adolescent populations that would be most appropriate for drug abuse prevention. Similarly, it is clear is that a number of different strategies are effective under controlled experimental conditions, but not clear which strategies (or package of strategies) will successfully translate from the laboratory to ‘live’ interventions producing long-term changes. In sum, a full implementation of an evidence-based DRD prevention program for individuals who are genetically at-risk for more impulsive discounting (and thereby addictive disorders) is simply not supported by current literature.

Where these links are weakest are the future priorities for the field. Progress in more definitively identifying genetic correlates of DRD is essential. Equally, a leading priority going forward is for pilot research to determine the utility of the discounting reduction strategies in adolescent samples to identify promising strategies for prevention contexts. Such studies would be well-suited to focus on proximal outcomes, most obviously DRD itself, drug-related motivation, and short-term substance abuse outcomes. The basis for presuming downstream positive effects of reducing DRD, of reorienting an individual away from immediate impulses and toward making more future oriented decisions, is a logical step forward. Developing efficacious interventions holds wide implications, not only for addictive disorders, but also more broadly on behaviors such as good nutrition, financial planning, and health maintenance behaviors that impact wide swaths of the general public (Howlett et al., 2008; Bradford, 2010; Epstein et al., 2010).

An important step in the future will be to identify standardized norms for DRD performance. As discounting is typically only assessed in research contexts, there is currently no basis for determining who should be targeted as a result of their DRD performance. In order to provide secondary prevention programming, it is necessary who is and who is not at-risk. The absence of normative data is a prosaic but nonetheless significant impediment to progress in this area.

The final point worthy of discussion is whether targeting discounting based on genetic profiles is a worthwhile undertaking more broadly. Certainly, from the perspective of personalized medicine, optimization of any approach using idiographic data (genetic or otherwise) is desirable. However, given the small and inconsistent relationships between risk alleles and impulsive DRD, as well as the extra step involved in genotyping individuals, a more feasible alternative would be using standardized normative data for risk identification rather than genetic risk profiles. In addition, it is also notable that psychosocial factors, such as early life stress, have also been associated with more impulsive discounting (e.g., Lovallo et al., 2013) and may be useful for risk identification. In other words, at the current time, there is a much stronger rationale for an efficacious DRD prevention program to be deployed for individuals who are in high-risk groups or exhibit DRD rates that significantly deviate from standardized norms than based on genotype. Alternatively, among young adults, the misuse of alcohol, tobacco, and other drugs is so prevalent, indeed almost normative in the case of alcohol, and the links between DRD and diverse forms of externalizing behavior are so robust, that primary prevention (i.e., universal) may be a more appropriate strategy. Intervention matching and secondary prevention are typically assumed to be desirable to maximize impact and efficiency, but, in this case, if an efficacious multi-component impulsive DRD prevention program can be developed, it will be of relevance to the large majority of young adults.

## Conclusion

The goal of this review was to evaluate the viability of DRD as a target for addictive disorders from the perspective of genetically-informed drug abuse prevention. A large body of research links impulsive DRD to drug abuse and supports the hypothesis that DRD is an endophenotype for addictive disorders. Additionally, current findings suggest that there are multiple promising methods—behavioral, pharmacological, and neuromodulatory—for acutely reducing DRD. However, the evidence for long-term changes and subsequent salutary health benefits is scant and no studies have directly assessed preventive interventions for impulsive DRD. Although significant gaps in knowledge remain and the wisdom of the long-term goal of genetically-informed drug abuse prevention via DRD is debatable, the current state of the science nonetheless suggests a more cautious conclusion, that impulsive DRD is more generally a promising target for drug abuse prevention and specific empirical investigations in this area are warranted.

### Conflict of Interest Statement

The authors declare that the research was conducted in the absence of any commercial or financial relationships that could be construed as a potential conflict of interest.
